# Porcine Sapovirus in Northern Vietnam: Genetic Detection and Characterization Reveals Co-Circulation of Multiple Genotypes

**DOI:** 10.3390/vetsci10070430

**Published:** 2023-07-01

**Authors:** Hieu Van Dong, Thai Ha Truong, Giang Thi Huong Tran, Witsanu Rapichai, Amonpun Rattanasrisomporn, Kiattawee Choowongkomon, Jatuporn Rattanasrisomporn

**Affiliations:** 1 Center for Advanced Studies for Agriculture and Food, Kasetsart University Institute for Advanced Studies, Kasetsart University, Bangkok 10900, Thailand; dvhieuvet@vnua.edu.vn; 2 Faculty of Veterinary Medicine, Vietnam National University of Agriculture, Trau Quy Town, Gia Lam District, Hanoi 131000, Vietnam; ththai@vnua.edu.vn (T.H.T.); tthgiang@vnua.edu.vn (G.T.H.T.); 3 Department of Companion Animal Clinical Sciences, Faculty of Veterinary Medicine, Kasetsart University, Bangkok 10900, Thailand; tswitsanu@gmail.com; 4 Department of Biochemistry, Faculty of Science, Kasetsart University, Bangkok 10900, Thailand; fsciktc@ku.ac.th; 5 Interdisciplinary of Genetic Engineering and Bioinformatics, Graduate School, Kasetsart University, Bangkok 10900, Thailand; fgraapr@ku.ac.th

**Keywords:** genotype, porcine sapovirus, sapovirus, pigs, Vietnam

## Abstract

**Simple Summary:**

This study investigated Sapovirus (SaV) infection in pigs farmed in northern Vietnam and conducted genetic characterization of the virus strains circulating in the country. In total, 102 samples were collected from piglets, fattening pigs, and sows with diarrhea in several cities and provinces in northern Vietnam. The porcine sapovirus (PoSaV) genome was examined using polymerase chain reaction. Sequencing of the partial RNA-dependent RNA polymerase (RdRp) gene region (324 bp) was performed. In total, 10 (9.8%) out of the 102 samples were positive for the PoSaV genome. Genetic analysis of the partial RdRp gene region indicated that the nucleotide identity of the 10 PoSaV strains obtained in this study ranged from 61.39% to 100%. Among the 10 strains obtained, 8 belonged to Genotype III and the remaining 2 strains were clustered in Genotype VIII.

**Abstract:**

Porcine sapovirus (PoSaV) has been reported in many countries over the world, which may cause gastroenteritis symptoms in pigs with all ages. There has been no report on PoSaV infection in Vietnam up to now. In this study, a total of 102 samples were collected from piglets, fattening pigs, and sows with diarrhea in several cities and provinces in northern Vietnam. The PoSaV genome was examined using polymerase chain reaction (PCR). Sequencing of the partial RNA-dependent RNA polymerase (RdRp) gene sequences (324 bp) was performed. Of the 102 tested samples, 10 (9.8%) and 7/20 (35%) were detected as positive for the PoSaV RdRp gene using the PCR method at the individual and farm levels, respectively. Genetic analysis of the partial RdRp gene region of about 324 bp indicated that the nucleotide identity of the current 10 Vietnamese viral strains ranged from 61.39% to 100%. Among the 10 strains obtained, 8 belonged to genotype III and the remaining 2 strains were clustered in genotype VIII. The Vietnamese genotype III viruses formed two sub-clusters. The Vietnamese PoSaV strains were closely related to PoSaVs reported in South Korea, Venezuela, and the Netherlands. This research was the first to describe PoSaV infection in northern Vietnam during 2022–2023.

## 1. Introduction

Sapoviruses (SaVs), having cup-shaped surface depressions, have been reported as human and animal pathogens. The viral infection may result in symptoms of gastroenteritis characterized by diarrhea or, less frequently, vomiting with diarrhea in their living specific hosts [1]. They were first reported in the United Kingdom in 1976 and Sapporo, a city of Japan, in 1977 from stool samples of children with gastroenteritis using electron microscopy by different scientific teams [2,3]. Thereafter, the porcine sapovirus (PoSaV) (Cowden strain) was firstly discovered after being isolated from a piglet in 1980 [4]. SaV infection has a huge spectrum of hosts, consisting of domestic pigs, dogs, bats, wild boars, chimpanzees, and humans [2,3,5,6,7,8,9]. Experimentally, SaV strains could result in clinical disease in piglets [10,11]. Flynn et al. (1988) used the SaV Cowden strain, which was passaged 12 times in cell culture, to inoculate orally in piglets at four days of age [11]. SaV-infected pigs developed the clinical sign of diarrhea at 3 days post infection. This sign was recorded during 3 to 7 days post-infection [11]. Viral shedding was detected in feces from SaV-infected pigs at 1 to 3 days post-inoculation until 30 ± 4 days after virus inoculation [10].

As a member of the genus Sapovirus within the family *Caliciviridae*, PoSaVs are a non-enveloped form. The viral genome consists of a single-stranded, positive sense RNA, which contains about 7.1 to 7.7 kb. RNA virus contains three open reading frames (ORFs) 1, 2, and 3. Among those, ORF 2 encodes a capsid protein, while the remaining ORF1 and 3 codes are for nonstructured NS1 to NS7 and small structural VP2 proteins. Among these genes, the capsid gene plays a critical role in the immunogenicity of the pathogen. A high genetic variability was reported among capsid gene sequences of Sapovirus strains. Therefore, ORF1 can be used for the genetic characterization of viral strains [2,12]. The VP2 and RNA-dependent RNA polymerase gene sequences were also used to characterize the viral strains, which was previously described in [13,14].

For classification, phylogenetic analysis of full-length capsid gene sequences indicated that PoSaV strains were genetically formed of four Genogroups III, VI, VII, and VIII in Japan, China, and Italy [9,15,16]. Reuter et al. conducted genetic analysis of the PoSaV strain detected in six European countries (Hungary, Denmark, Finland, Italy, Spain, and Slovenia) during 2004–2007 [17]. Genogroups III, VI, and VIII PoSaV viruses were reported among pigs in these six countries. In addition, the authors pointed out that the two new Genogroups IX and X were found in Denmark, Finland, Spain, Italy, and Slovakia [17]. Enteric caliciviruses are crucial for maintaining public health since approximately 23 million infection cases were yearly reported as foodborne illness in humans [18]. Furthermore, some caliciviruses can spread to animals [3,14,19]. Although evidence of transmission of swine in human Sapoviruses remain unclear, the likelihood that they act as a reservoir species is increased by the prevalence of infection and recombination in pigs [6,20,21,22]. However, there have never been any proven instances of PoSaVs spreading from pigs to humans.

In Vietnam, several studies have reported on Sapovirus in humans. In both northern and southern Vietnam, SaVs were detected in acute gastroenteritis-suspected children [23,24]. Nguyen et al. (2008) reported that six (0.99%) samples from child patients during 2005 to 2006 showed acute gastroenteritis in Ho Chi Minh city, a big city of southern Vietnam [24]. Phylogenetic analysis, based on the partial capsis gene (434 bp) sequence, indicated that the six SaV strains belonged to genotype I.1 (2 strains), I.2 (1 strain), II.1 (1 strain), and II.4 (2 strains) [24]. From 2007 to 2008, 501 fecal samples were collected from children (aged < 5 years) indicating acute gastroenteritis in Hanoi, Vietnam. Genetic analysis indicated that seven (1.4%) samples were positive for the SaV genome using the polymerase chain reaction (PCR). All seven SaVs that were obtained belonged to genotypes I and II [23]. Few studies have been reported regarding the presence of SaVs in animals in the country. Here, we first reported the incidence of PoSaV strains in pigs raised in several cities and provinces in northern Vietnam. This study should enhance our understanding of molecular characterization among PoSaVs in Vietnam.

## 2. Materials and Methods

### 2.1. Ethics Statement

This research did not contain any studies involving human participants. Fecal samples were collected from pigs under the auspices of the Vietnam National University of Agriculture. The protocol for sampling was approved by the Committee on animal reseach and ethics of the university (CARE-2022/08). Sampling was also permitted by the pig farm owners.

### 2.2. Samples

**Figure 1:**
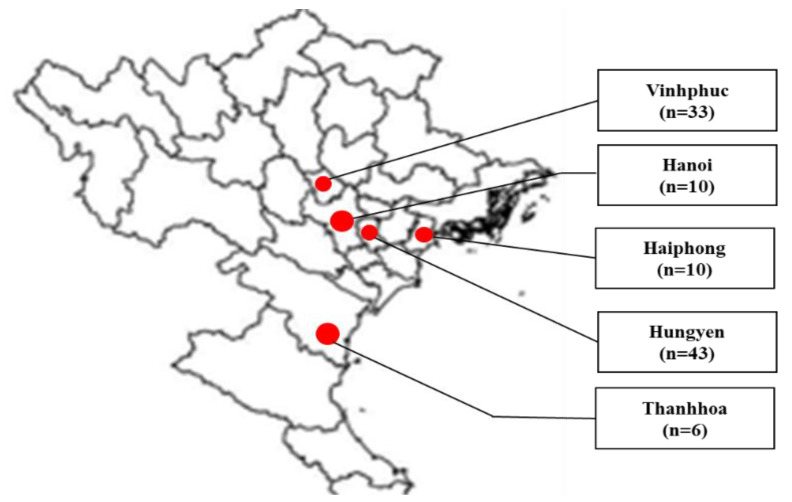
Map of sampling areas in northern Vietnam. Cities and provinces for sampling are marked at red circles. Fecal samples were collected from Vinhphuc (33), Hanoi (10), Haiphong (10), Hungyen (43), and Thanhhoa (6).

In total, 102 fecal samples were collected from piglets, fattening pigs, and sows from several cities or provinces in northern Vietnam, consisting of Hanoi (*n* = 10), Haiphong (*n* = 10), Hungyen (*n* = 43), Thanhhoa (*n* = 6), and Vinhphuc (*n* = 33) during July 2022 to March 2023 (Figure 1). Pigs showing diarrhea and dehydration were selected for sample collection. From each farm, 3 to 6 pigs were selected for sampling. Fecal samples were kept in homogenized phosphate-buffered saline. The homogenate was prepared at 10% and kept at −80 °C until use.

### 2.3. Total RNA Extraction and cDNA Synthesis

A GeneAll^®^ Ribospin vRD II Kit (GeneAll Biotechnology; Gyeonggi-do, Republic of Korea) was used to extract the total RNA from the field samples. Extraction protocol was performed following instructions of the manufacturer. RNA was dissolved in 50 µL of distilled water and kept in −80 °C until use.

To synthesize cDNA, a total of 20 µL reagents containing 4 µL of 5X M-MLV buffer, 1 µL of random primer (Invitrogen; Carlsbad, CA, USA), 1 µL of dNTP (10 mM), 1 µL of DTT, 1 µL of M-MLV reverse transcriptase (Invitrogen), 8 µL of distilled water, and 4 µL of RNA were placed in a thermal PCR machine under condition at 25 °C/10 min, 37 °C/1 h, and 65 °C/10 min. cDNA was directly used for PCR or preserved at −30 °C until use.

### 2.4. PCR and Nucleotide Sequencing

PCR was conducted to detect the SaV RNA-dependent RNA polymerase (RdRp) gene, group A porcine rotavirus (PoRV), porcine epidemic diarrhea virus (PEDV), and Transmissible gastroenteritis virus (TGEV) genome in the field samples. Four sets of primers (P289/P290, Rot3/Rot5, P1/P2, and T1F/T2R) were used in this study, as indicated in Table 1, previously reported in [25,26,27]. The target amplification was a partial RdRp gene region, as previously described [27]. A volume of 25 µL PCR reagents, consisting of 12.5 µL of GoTag^®^ Green Master Mix (Promega), 1 µL of each forward and reverse primer (10 mM), 10.5 µL of distilled water, and 2 µL of cDNA as templates, were conditioned thermally at 94 °C/5 min, 35 cycles of 94 °C/30 s, 49 °C to 53 °C (depends on primers)/60 s, and 72 °C/2.5 min, followed by extension at 72 °C/10 min. A 1.2% agarose gel was used to perform electrophoresis of the PCR product of 331 bp, which was captured under ultraviolet radiation. PCR products were purified by using a GeneClean^®^ II Kit (MP Biomedicals; Santa Ana, CA, USA). The protocol was followed by the manufacturer’s instructions. The PCR-purified products were then sent to the 1st BASE company (Malaysia) to sequence.

### 2.5. Data Analysis

Alignment and analysis of sequence data were performed using the Clustal W [28] supplemented in the BioEdit software [29]. Afterward, the GENETYX version 10.0 (GENETYX Corp.; Tokyo, Japan) and the Basic Local Alignment Search Tool (BLAST) (https://blast.ncbi.nlm.nih.gov/ accessed on 2 May 2023) were used to examine the nucleotide identity among the current viral sequences and retrieved sequences from GenBank. Construction of a phylogenetic tree based on nucleotide sequences of the current Vietnamese PoSaV strains and 29 SaVs (Information of reference sequences is provided in Table 2) from GenBank was performed using a neighbor-joining method based on the Tamura-2 parameter model. The confidence values of branches on the phylogenetic tree were assessed based on bootstrapping with 1000 replicates using the MEGA X software (https://megasoftware.net/ accessed on 2 May 2023). The obtained sequences were deposited into GenBank. Accession numbers of the Vietnamese PoSaV sequences used in this study were OQ953988 to OQ953997.

### 2.6. Statistical Analysis

Significant differences in the percentages of SaV genome identification among the collected samples based on region, age, and scale of farm were detected by using Fisher’s exact test. A *p*-value < 0.05 was regarded as statistically significant.

## 3. Results

### 3.1. Identification of the PoSaV RdRp Gene in Fecal Samples

In the present study, fecal samples were collected from clinical pigs with diarrhea and dehydration. First of all, the percentages of SaV infection were detected using the PCR method. Among the tested samples, 10 (9.8%) were positive for the SaV genome using the reverse-transcription PCR method. The PEDV, TGEV, and porcine rotavirus genomes were not detected in these 10 SaV genome-positive samples (data not shown) by using conventional PCR. Within the studied regions, the highest positive rate was recorded in Hungyen province (13.95%), followed by Hanoi (10%), Haiphong (10%), and Vinhphuc (6.06%), while no sample (0/6) was positive for the viral genome in Thanhhoa province (Table 2). Of the 18 tested pig farms, 7 were positive for the SaV genome (Table 2). Positive rates in Hanoi and Haiphong were 50% and 50%, which were significantly (*p* < 0.01) greater than that in Vinhphuc (28.57%). The difference was not significant for the positive rates in Vinhphuc (28.57%) and Hungyen (37.5%) (Table 3).

The percentage of SaV-positive fattening pigs was 15.15%, followed (*p* > 0.05) by sows (10%), and ≥21-day-old piglets (6.06%), whereas no sample was positive for the SaV RdRp gene in <21-day-old piglets. The PoSaV-positive percentages for the scaled farms (<100, 100–300, and >300 individuals) were 5.77%, 15.00%, and 13.33%, respectively; however, these were insignificantly different (Table 4).

### 3.2. Genetic and Phylogenetic Characterization of the Vietnamese PoSaV Strains

Ten SaV genome-positive samples were forwarded for nucleotide sequencing. Sequence data from Sanger sequencing were used for the genetic characterization of the PoSaV-positive samples obtained from the pigs. Viral strains were designated as Vietnam/Pig/VNUA-P01, -P07, -P09, -P15, -P25, -P65, -P79, -P89, -P113, -P118/2002. Among the Vietnamese PoSaV strains, the nucleotide identity ranged from 61.39% (Vietnam/Pig/VNUA-P113/2022 vs. Vietnam/Pig/VNUA-P01/2022; Vietnam/Pig/VNUA-P118/2022 vs. Vietnam/Pig/VNUA-P01/2022) to 100% (Vietnam/Pig/VNUA-09/2022 vs. Vietnam/Pig/VNUA-25/2022). These 10 Vietnamese PoSaV strains were compared with other viral strains downloaded from GenBank. The highest nucleotide identity varied among strains: 86.62% (Vietnam/Pig/VNUA-P113/2022 vs. Netherlands/SWECII/VA103; Vietnam/Pig/VNUA-P118/2022 vs. Netherlands/SWECII/VA103), 90.63% (Vietnam/Pig/VNUA-P07/2022 vs. South Korea/Pig/JB-GC 90/04), and 92.65% (Vietnam/Pig/VNUA-P01/2022, -P09, P15, -P25, -P65, -P79, -P89 vs. Venezuela/Pig/Po/SV/Aragua/2003/VE) (Table 5).

**Figure 2:**
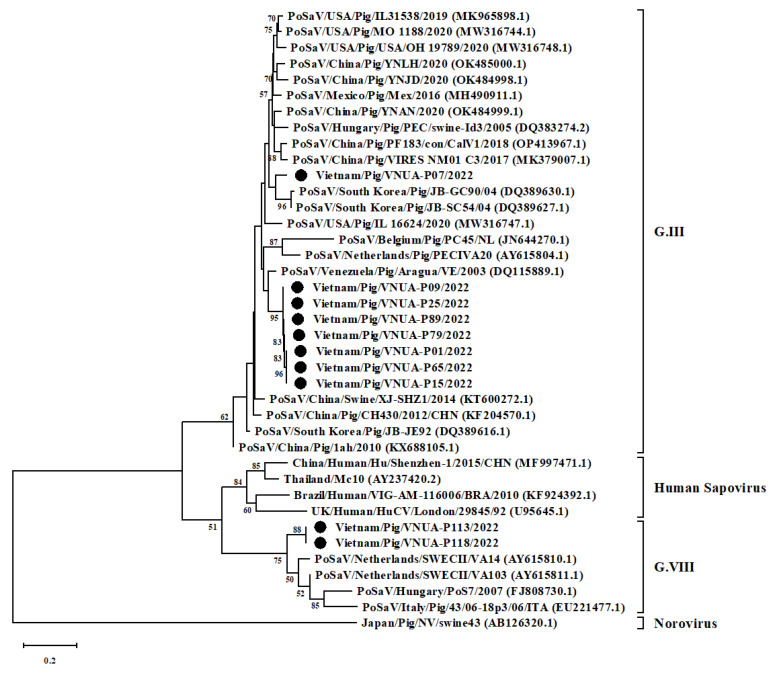
Neighbor-joining phylogenetic tree of RNA-dependent RNA polymerase gene (324 bp) sequences of current Vietnamese Porcine sapovirus (PoSaV) strains and other sequences downloaded from GenBank. Neighbor-joining method in MEGA X software (https://megasoftware.net/ accessed on 2 May 2023) was used with 1000 bootstrap replicates. Ten Vietnamese PoSaV strains were indicated by black circles.

The phylogenetic tree, constructed by using the partial RdRp gene sequences (324 bp) of 10 Vietnamese PoSaVs and 29 other sequences from GenBank, revealed that the current 10 Vietnamese PoSaV strains belonged to several lineages. Eight strains belonged to genotype III, while the remaining two strains were in genotype VIII. Among the eight PoSaV strains obtained, seven strains formed into a single group, which was separated from the remaining single strain (Vietnam/Pig/VNUA-P07/2022). The 10 PoSaV strains obtained were genetically related to Korean (JB-GC90/04 (GenBank accession number: DQ389630.1) and JB-SC54 strain (DQ389627.1)), Venezuelan (Aragua/VE (DQ115889.1), and the Netherlands (SWECII/VA14 (AY615810.1) and SWECII/VA103 (AY615811.1)) strains (Figure 2).

## 4. Discussion

SaV has been reported in both humans and animals [30]. To date, the virus has been distributed worldwide [12]. SaVs have been detected not only in humans but also in a variety of animals [2,3,5,6,7,8,9]. No direct evidence has been reported on whether or not SaV agents are a source of the disease in humans. Since reservoirs of SaV have been reported in many animals, understanding animal SaV strains may provide important information for human health. In Vietnam, SaVs have been reported in children with acute gastroenteritis [23,24]; however, there have been no published reports regarding its presence in animals in the country. The current study was the first to report PoSaVs circulating among pigs farmed in northern Vietnam. Only 10 (9.8%) were detected to be positive for the PoSaV RdRp gene using PCR. In addition, genetic characterization of the PoSaV strains was also conducted based on the partial RdRp sequences in the country. Regarding the rate of infection, SaVs might be detected in pigs at low (2.39% to 10.2%) [13,31] and high rates (43.1% in asymptomatic pigs and 45.3% in diarrhoeic pigs) [20]. In this study, the positive rate was 9.8%, which was similar to that of positive rates reported in China (9%) [32], the Czech Republic (10.2%) [13], and Slovakia (clinical healthy pigs: 8.4%; diarrhoeic pigs: 10%) [33]. The currently positive rate was lower than that of positive rates reported in the USA (62%) [34], Yunnan city of China (35.2%) [21], and Brazil (23.7%) [35]. The differences in percentage rates might have been due to the different locations and times of sampling. The current findings suggested that SaVs have been circulating and affecting pig production across Vietnam.

PoSaV was first recognized in the USA in 1980 in pigs coinfected with viruses [4]. The viral infection was clarified to be the result of gastroenteritis in both humans and animals [30]. Later researchers reported PoSaV strains in diarrhoeic and asymptomatic pigs [36,37]. In experimental piglets, PoSaV strains that had been recovered from cell culture successfully established symptoms of diarrhea and enteritis in the infected pigs [11,38]. The current study was the first to report PoSaV infection in pigs with diarrhea. Therefore, it must be considered that this virus is a diarrhoeic syndrome in pigs in the country. In addition, PoSaVs were reported in infected pigs of all ages. Early stages may be suitable for viral infection rather than other age groups [17,34].

It has been reported that PoSaV infection was detected in diarrhoeic pigs or piglets coinfected with other viruses, such as rotavirus [4], astrovirus [4], PEDV [39], porcine sapelovirus (PSV), porcine kobuvirus (PkoV), porcine torovirus (PTOV), and TGEV [40]. Chen et al. (2018) reported that the positive rate was 13% (28/217) of diarrhoeic piglets co-infected with PoSaV and PEDV in the USA during 2015 to 2016 [39]. Later, Shi et al. (2021) used a novel Luminex xTAG method to detect co-infections of several enteric viruses in pigs. The authors found that co-infections of PoSaV and PKoV, PoSaV and PEDV, PoSaV and PSV, and PoSaV and PTOV were about 11%, 1%, 2%, and 1.5%, respectively [40]. These findings suggested that dual infections of PoSaVs and other enteric viruses were common in diarrhoeic pigs, which might cause clinical symptoms. In the present study, we examined the genome of PEDV, TGEV, and Rotavirus in the field samples. The results confirmed that no co-infection of PoSaVs and the three tested enteric viruses. In addition, other bacterial and parasite pathogens were also found in suckling and weaned pigs with diarrhea in Japan, such as *Escherichia coli* and coccidian [41]. Therefore, other enteric viruses, bacterial, and parasite pathogens should be tested to detect the co-infections in pigs in Vietnam. In addition, sample sizes should be increased to further research co-infection of PoSaVs and other pathogens in pigs in the country.

In terms of SaV classification, several studies used the partial RdRp and partial VP1 gene sequence to characterize and classify the viral field strains [1,14,30]. Later researchers, using genetic characterization, reported differences based on the RdRp and VP1 regions [16,22]. Among SaV strains, Scheuer et al. (2013) reported 17 genotypes (II, III, and V–XIX) in animals and four genotypes (I, II, IV, and V) in humans [42]. Worldwide, at least eight genotypes (III and V–XI) have been reported in pigs [12,30]. In addition, co-circulation of highly divergent PoSaV strains, belonging to genotypes III and VI was found among pigs in the USA [16]. The current study identified 10 PoSaV strains belonging to genotypes III (eight strains) and VIII (two strains). Among those strains, genotype III PoSaV viruses, divided into two subclusters, were the most dominant circulation. Co-circulation of both genotypes III and VIII PoSaV strains reflected that the virus may have highly divergent circulation in northern Vietnam.

Recombination is defined as an evolutionary process. Globally, recombination has been found among SaV strains [6,22]. The recombination may result in the generation of novel SaV strains and novel genotypes [6,22]. Due to recombination events, divergence among SaV strains was detected using the VP1 gene and partial RdRp sequences [1]. In the current study, co-circulation of the two genotypes III and VIII of PoSaV strains were found, which could provide chances for generating recombination events and novel PoSaV strains. A small region of the RdRp gene of PoSaV strains were sequenced and used to characterize the viral strains in Vietnam. Further research should be conducted to sequence the full-length VP1 and RdRp gene sequences of the present PoSaV strains in the country.

Previous studies reported that PoSaV may be a potential zoonotic pathogen. Firstly, Costantini et al. (2006) noted that co-infection of human SaV and PoSaV was found [19]. Later, recombination events Occurred between PoSaV and human SaV strains to generate a novel viral strain [15,16]. Among PoSaV strains, genotype V.3 and V.5 viruses were most genetically related to human Sapoviruses [12]. To our knowledge, no direct evident of zoonotic transmission between PoSaV and human Sapovirus has been reported. In the present study, only genotypes III and VIII strains were reported among Vietnamese PoSaV strains, but these viruses were not clustered with human Sapovirus. In addition, this analysis was based only on the partial RdRp gene sequence (324 bp). Therefore, the current results were not sufficient to conclude that zoonotic transmission occurred among viral strains. Further genetic analyses should be conducted based on the complete genome and full-length RdRp, VP1, or VP2 gene sequences.

## 5. Conclusions

The present study was the first to report PoSaV infection in 10 clinically diarrhoeic pigs in northern Vietnam. Molecular analysis of the partial RdRp gene sequences of the Vietnamese PoSaV strains revealed that the ten viral strains were genotypes III and VIII viruses. The present Vietnamese PoSaV strains obtained in this study were genetically close to the PoSaV strains reported in several countries, suggesting that since PoSaVs are not a pathogen evaluated in pig imports, the movement of high-genetic value animals to these countries could have spread some strains around the world. Co-circulation of the two genotypes was detected and this characteristic should be involved in the establishment of prevention strategies to control this pathogen in the country.

## Figures and Tables

**Table 1:** Primers used for PCR in this study.

Target Virus	Name of Primer	Nucleotide Sequence (5′–3′)	PCR Product (bp)	References
Sapovirus	P289	TGA CAA TGT AAT CAT CAC CAT A	331	[27]
	P290	GAT TAC TCC AAG TGG GAC TCC AC		
Porcine rotavirus	Rot3	AAA GAT GCT AGG GAC AAA ATT G	308	[25,26]
	Rot5	TTC AGA TTG TGG AGC TAT TCC A		
Porcine epidemic diarrhea virus	P1	TTC TGA GTC ACG AAC AGC CA	650	[26]
	P2	CAT ATG CAG CCT GCT CTG AA		
Transmissible gastroenteritis virus	T1F	GTG GTT TTG GTY RTA AAT GC	859	[26]
	T1R	CAC TAA CCA ACG TGG ARC TA		

**Table 2:** Description of Sapovirus and Norovirus strains used in this study.

GenBank Accession No.	Strain	Location	Source	Year	Virus
KX688105.1	1ah	China	Pigs	2010	Sapovirus
KF204570.1	CH430	China	Pigs	2012	Sapovirus
KT600272.1	XJ-SHZ1	China	Pigs	2014	Sapovirus
MF997471.1	Shenzhen-1	China	Human	2015	Sapovirus
MK379007.1	IRES_NM01_C3	China	Pigs	2017	Sapovirus
OP413967.1	PF183	China	Pigs	2018	Sapovirus
OK485000.1	YNLH	China	Pigs	2020	Sapovirus
OK484998.1	YNJD	China	Pigs	2020	Sapovirus
OK484999.1	YNAN	China	Pigs	2020	Sapovirus
KF924392.1	VIG-AM-116006/BRA	Brazil	Human	2010	Sapovirus
AY237420.2	Mc10	Thailand	Human	2003	Sapovirus
U95645.1	29845	UK	Human	1992	Sapovirus
EU221477.1	43/06-18p3/06/ITA	Italy	Pigs	2006	Sapovirus
JN644270.1	PC45/NL	Belgium	Pigs	2009	Sapovirus
DQ389630.1	JB-GC90	Republic of Korea	Pigs	2004	Sapovirus
DQ389627.1	JB-SC54	Republic of Korea	Pigs	2004	Sapovirus
DQ389616.1	JB-JE92	Republic of Korea	Pigs	2005	Sapovirus
MK965898.1	IL31538	USA	Pigs	2019	Sapovirus
MW316744.1	MO_1188	USA	Pigs	2020	Sapovirus
MW316748.1	OH_19789	USA	Pigs	2020	Sapovirus
MH490911.1	Mex	Mexico	Pigs	2016	Sapovirus
DQ115889.1	Aragua	Venezuela	Pigs	2003	Sapovirus
MW316747.1	IL_16624	USA	Pigs	2020	Sapovirus
AY615811.1	SWECII/VA103	The Netherlands	Pigs	2004	Sapovirus
AY615810.1	SWECII/VA14	The Netherlands	Pigs	2004	Sapovirus
AY615804.1	PECIVA20	The Netherlands	Pigs	2004	Sapovirus
DQ383274.2	PEC/swine-Id3	Hungary	Pigs	2005	Sapovirus
AB126320.1	swine43	Japan	Pigs	2003	Norovirus

**Table 3:** Detection of the Sapovirus RdRp gene in pigs in the field in different cities and provinces in northern Vietnam using PCR method.

Province/City	No. of Collected Samples	No. of Gene-Positive Samples/(%)	No. of Farms	No. of Gene-Positive Farms/(%)
Hanoi	10	1/(10.00) ^x^	2	1/(50.00) ^x^
Haiphong	10	1/(10.00) ^x^	2	1/(50.00) ^x^
Hungyen	43	6/(13.95) ^x^	8	3/(37.50) ^xy^
Thanhhoa	6	0/(0.00)	1	0/(0.00)
Vinhphuc	33	2/(6.06) ^x^	7	2/(28.57) ^y^
Total	102	10/(9.80)	20	7 (35.00)

Different lowercase superscripts ^x^, ^y^ indicate significantly different between groups (*p*-value < 0.05).

**Table 4:** Detection of Sapovirus genome in fecal samples of pigs according to age and scale of farm.

Type of Pigs/Farm Scale	Age	Number of Tested Samples	Number of RdRp Gene-Positive Samples/(%)
Piglets	<21 days	6	0/(0.00)
	≥21 days	33	2/(6.06)
Fattening pigs		33	5/(15.15)
Sows		30	3/(10.00)
<100		52	3/(5.77)
100–300		20	3/(15.00)
>300		30	4/(13.33)

**Table 5:** Comparisons of nucleotide identity of the partial RNA-dependent-RNA polymerase gene (324 bp) sequences of the current Vietnamese PoSaV strain with that of retrieved sequences from GenBank.

Strain Name	Virus with the Highest Nucleotide Identity				
	Strain	Country	Accession Number	Year	% Identity
Vietnam/Pig/VNUA-P01/2022	Aragua/VE	Venezuela	DQ115889.1	2003	92.65
Vietnam/Pig/VNUA-P07/2022	JB-GC90/04	Republic of Korea	DQ389630.1	2004	90.63
Vietnam/Pig/VNUA-P09/2022	Aragua/VE	Venezuela	DQ115889.1	2003	92.65
Vietnam/Pig/VNUA-P15/2022	Aragua/VE	Venezuela	DQ115889.1	2003	92.65
Vietnam/Pig/VNUA-P25/2022	Aragua/VE	Venezuela	DQ115889.1	2003	92.65
Vietnam/Pig/VNUA-P65/2022	Aragua/VE	Venezuela	DQ115889.1	2003	92.65
Vietnam/Pig/VNUA-P79/2022	Aragua/VE	Venezuela	DQ115889.1	2003	92.65
Vietnam/Pig/VNUA-P89/2022	Aragua/VE	Venezuela	DQ115889.1	2003	92.65
Vietnam/Pig/VNUA-P113/2022	SWECII/VA103	The Netherlands	AY615811.1	2016	86.62
Vietnam/Pig/VNUA-P118/2022	SWECII/VA103	The Netherlands	AY615811.1	2016	86.62

## Data Availability

The datasets used and/or analyzed during the current study are available from the corresponding author on reasonable request.
